# Binding Characteristics Study of DNA based Aptamers for *E. coli* O157:H7

**DOI:** 10.3390/molecules26010204

**Published:** 2021-01-03

**Authors:** Saika Siddiqui, Jie Yuan

**Affiliations:** 1 Department of Bioengineering, Hong Kong University of Science and Technology, Kowloon, Hong Kong; 2 Department of Electronic and Computer Engineering, Hong Kong University of Science and Technology, Kowloon, Hong Kong; eeyuan@ust.hk

**Keywords:** DNA aptamers, *E. coli* O157:H7 detection, aptamer binding characteristics, biotin modification, ionic buffer strength

## Abstract

*E. coli* O157:H7 is a pathogenic bacterium producing verotoxins that could lead to serious complications such as hemolytic uremia syndrome. Fast detection of such pathogens is important. For rapid detection, aptamers are quickly gaining traction as alternative biorecognition molecules besides conventional antibodies. Several DNA aptamers have been selected for *E. coli* O157:H7. Nonetheless, there has not been a comparative study of the binding characteristics of these aptamers. In this work, we present a comprehensive analysis of binding characteristics including binding affinity (K_d_) and binding capacity (B_max_) of DNA-based aptamers for *E. coli* O157:H7 using qPCR. Our results show that aptamer E18R has the highest binding capacity to *E. coli* 157:H7 and the highest specificity over non-pathogenic *E. coli* strains K12 and DH5α. Our study also finds that the common biotin-tag modification at 5′ end typically changes the binding capacity significantly. For most of the selected aptamers, the binding capacity after a biotin-tag modification decreases. There exists a discrepancy in the binding capability between the selected aptamer and the aptamer used for detection. Our study also shows that a lower concentration of Mg^2+^ ions in the binding buffer leads to a decrease in the binding capacity of E17F and E18R, while it does not affect the binding capacity of S1 and EcoR1.

## 1. Introduction

Escherichia coli (*E. coli*) is a gram-negative bacterium which forms the normal flora in human and animal gastrointestinal tracts [1]. While most strains are harmless, some strains could be harmful such as Shiga toxin producing *E. coli* (STEC), of which *E. coli* O157:H7 is the most important serotype due to its involvement in several outbreaks [2]. *E. coli* O157:H7 produces verotoxins that cause severe damage to the lining of the intestines, leading to bloody diarrhea. In more vulnerable groups, such as young children and elderly, verotoxins could develop more serious conditions as hemolytic uremia syndrome (HUS) [3,4]. According to the WHO, 10% of people infected with STEC could develop HUS.

Although antibodies have been widely used in the detection of pathogens, the need for a portable, fast and low-cost detection instrument has directed research in recent years towards exploring alternative biorecognition molecules like aptamers. Aptamers are oligonucleotides, DNA or RNA, capable of binding to target molecules based on their nucleic acid sequences that fold into the three-dimensional structures. The three-dimensional structure is also dependent on the binding conditions used (e.g., temperature and ionic concentration) [5,6,7]. The use of aptamers as alternatives to antibodies in biorecognition and detection has grown rapidly due to their advantages, such as ease of synthesis, low cost, no or minimum variation among batches and long shelf life. Aptamers are also easy to modify chemically, making them more versatile [8].

Using the SELEX technique [9], aptamers for various pathogens have been actively searched for in recent years [10,11,12]. Active research has been performed for *E. coli* O157:H7 as well [13,14,15]. Besides the search, some of these studies also incorporated the selected aptamers with biosensors based on different sensing principles such as quartz crystal microbalance (QCM), fluorescent based detection using quantum dots [14,15]. Most of these studies have either focused on the selection of aptamers or their application in biosensors. None of them has systematically studied the binding characteristics of the selected aptamers.

The dissociation constant (binding affinity-K_d_) and the maximum binding capacity (B_max_) are the two most important parameters for quantifying the aptamers binding to a target [16]. Their characterization relies on the precise measurement of the associated concentrations. Different methods have been applied to perform such measurement, such as flow cytometry [13], quantitative PCR [17], fluorescent spectroscopy [18], Enzyme Linked Oligonucleotide Assay (ELONA) [19] or custom designed biosensors [14]. Also, these binding characteristic measurements were often performed under different conditions that could lead to analytical difficulties in their application into other biosensors [20]. Aptamer binding is sensitive to the ionic environment [21,22]. Importantly, in most of these measurements, aptamers were attached to labels for quantification. As the binding is sensitive to the aptamer structure, the labels could affect the measurements significantly [23,24]. Thus, the reported binding characteristics vary significantly. In this work, we perform a systematic characterization of DNA-based aptamers for *E. coli* O157:H7 based on real time or the quantitative polymerase chain reaction (qPCR). The study included all major *E. coli* O157:H7 DNA aptamers reported in the literature AM6 [13], S1 [14], EcoR1 [15], E17F, and E18R [25]. E17F and E18R have also been used to develop biosensors [26,27,28]. The list of DNA based aptamers used in this study along with their reported K_d_ values for *E. coli* O157:H7 are given in Table 1. Quantitative PCR (qPCR) is a simple, sensitive and quantitative technique and is considered the golden standard in quantitative analysis of nucleic acids [29]. The experiments were performed under uniform condition. The effect of biotin-tag modification on the 5′ end of the aptamer on binding characteristics of the aptamers was studied. The effect of monovalent and divalent cations, K^+^ and Mg^2+^, in the binding buffer on the binding characteristics of these aptamers was also studied. The nonspecific binding characteristics of these aptamers against other non-pathogenic strains of *E. coli* (K12 and DH5α) were also investigated. We believe this study provides a clearer picture about the binding performance of aptamers with *E. coli* O157:H7. This study could be important in the development of new aptamers and their application to biosensors. 

## 2. Results and Discussion

### 2.1. Qualitative Analysis

**Figure 1:**
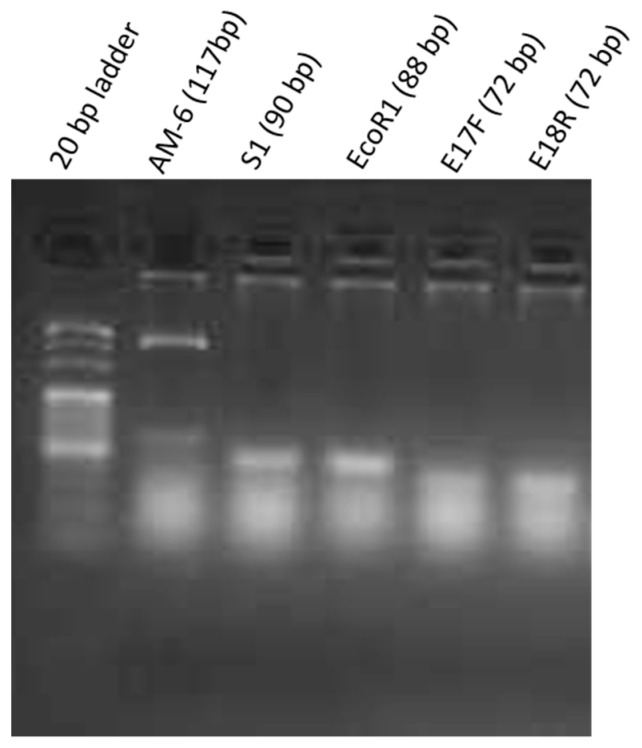
Agarose gel electrophoresis (1.5%) of aptamer amplicons by PCR lane 1—20 bp DNA marker, lane 2—AM6 aptamer, lane 3—S1 aptamer, lane 4—EcoR1 aptamer, lane 5—E17F aptamer, lane 6—E18R aptamer.

After the qualitative PCR experiment, S1, EcoR1, E17F, and E18R showed single amplification bands of 90 bps, 80 bps, 72 bps and 72 bps respectively on 1.5% agarose gel after electrophoresis. However, the amplification product of AM-6 showed an additional band at 500 bps as shown in Figure 1. A possible explanation is that the primers of AM-6 amplify *E. coli* genomic DNA of around 500 bps. In order to confirm the binding of AM-6 primers to bacterial genomic DNA, negative control experiments as described in Section 3.3 were performed without the step of cell-aptamer binding. By performing PCR with selected primers on this binding-free assay, it is expected that no significant band can be found in the gel electrophoresis as no bound aptamers should be present in these assays. This is true for all listed aptamers except AM-6, which confirmed the nonspecific amplification of genomic DNA of *E. coli* O157:H7 with AM-6 primer. Hence, aptamer AM-6 was excluded from further analysis. RS also showed an amplification band suggesting some degree of binding to bacterial cells.

Minimum free energy structure of the aptamers predicted by mfold software are given in Supplementary Figure S1 (binding buffer ionic conditions at 37 °C). Similar structures have also been reported by refs. [13,14,15]. The difference in the minimum free energy of all the aptamers is not very considerable. Additionally, sequence homology analysis did not show any conservative motifs in all the aptamers (Supplementary Figure S2). All the aptamers present bulges and hairpin loop structures.

### 2.2. Quantitative Analysis (K_d_ Analysis by qPCR)

The quantitative study was performed by qPCR. As opposed to Scathard plots, non-linear regression was used to measure K_d_ by fitting the data to the equation below [16]:

$$\left[\mathrm{BA}\right]=\frac{\left[A\right]B_{\max}}{K_{d}+\left[A\right]}$$

where B_max_ is defined as the maximum binding capacity when all receptors are fully saturated with aptamers. [BA] is the concentration of the bound aptamer, while [A] is the incubated aptamer concentration.

#### 2.2.1. Quantitative Binding Analysis with *E. coli* O157:H7

**Figure 2:**
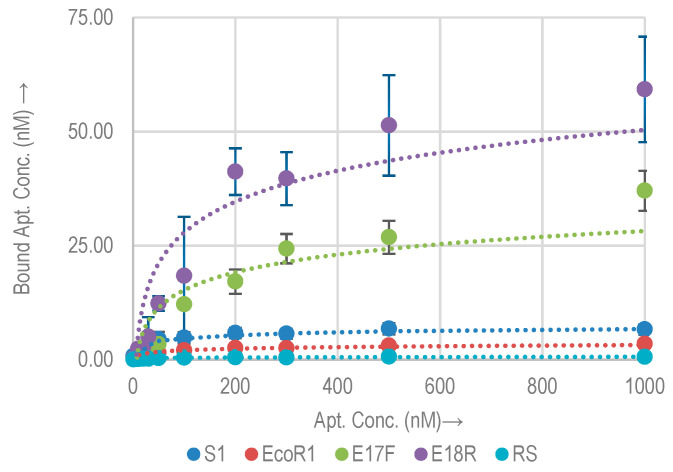
Binding isotherms for aptamers and random sequence (RS) with *E. coli* O157:H7.

The binding affinity and capacity of the selected aptamers (S1, EcoR1, E17F, and E18R) with *E. coli* O157:H7 (≈10^8^ cells) were measured. Aptamer concentrations ranging from 0.1 nM to 1000 nM were incubated with *E. coli* O157:H7. Figure 2 shows the binding isotherms of the selected aptamers for target *E. coli* O157: H7. The K_d_ and B_max_ values, as derived from the fitting curve technique are listed in Table 2. Compared to the random sequence control, all the aptamers showed higher binding to the bacterial cells. The random sequence has a B_max_ of 0.6 nM. This confirms that the binding of the aptamers to the target is based on the formation of secondary structures. Approximate binding sites per bacterial cell were calculated using the B_max_ value and are listed in Table 2. Among the selected aptamers, E18R exhibited the highest B_max_ (=54.3 nM). The lowest value is shown by EcoR1 (=3.4 nM). As compared to the random control sequence, these values are higher by a factor of approx. 85× and 5×, respectively. The relative B_max_ as compared to the control for the rest of the aptamers are listed in Table 2. S1 shows the lowest K_d_ of 25.7 nM, while E18R has the highest K_d_ of 151 nM. On average, an *E. coli* O157:H7 cell has thousands of binding sites for these aptamers.

Each of these sites may possess a different binding affinity. Thus a higher B_max_ does not necessarily translate into a higher binding affinity (low K_d_), and vice versa [17,30]. E18R shows the largest amount of binding sites on the cell, which could correspond to the strongest signal for biosensors. EcoR1 shows the least amount of binding sites. 

In [14], K_d_ of S1 was measured to be 10.3 nM based on QCM, which is approximately 2.3× lower than our measurements. Nonetheless, measurements in [14] were made on different QCM sensors due to the reusability issue, which showed larger variation range than our measurements. Our measurements based on qPCR have a smaller error margin. 

#### 2.2.2. Non-Specific Aptamer Binding Analysis with *E. coli* K12 and DH5α

**Figure 3:**
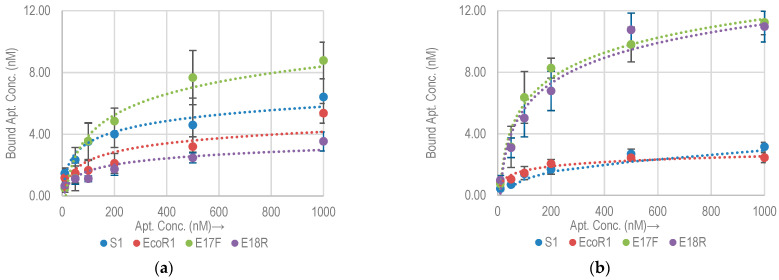
Binding isotherms for four DNA based aptamers with (**a**) *E. coli* K12 and (**b**) *E. coli* DH5α.

**Figure 4:**
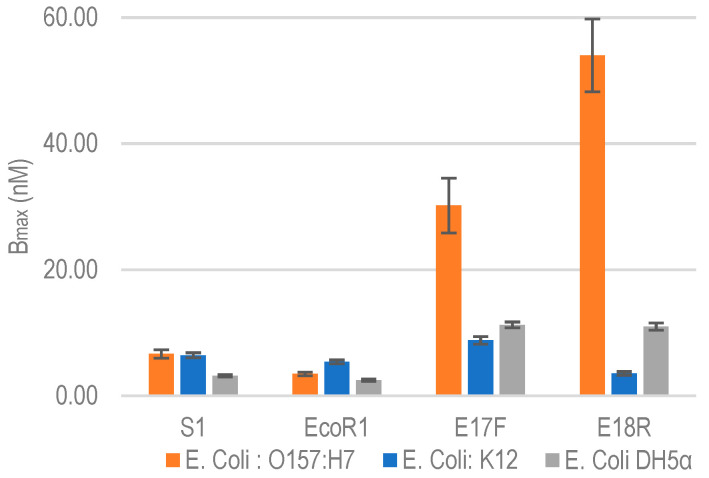
Relative B_max_ comparison against non-specific *E. coli* bacterial strains. Data represent Mean ± SD of 3 independent experiments.

To evaluate the specificity of selected aptamers against nonspecific bacterial strains, we tested their cross reactivity by performing binding assay against *E. coli* K12 and *E. coli* DH5α. Both are commonly used non-pathogenic lab strains of *E. coli*. Figure 3 shows the binding isotherms of aptamers against *E. coli* K12 and DH5α. The B_max_ of each of the aptamers for *E. coli* K12 and DH5α along with approximate binding sites per bacterial cell are listed in Table 3. Comparative B_max_ for *E. coli* O157:H7, K12 and DH5α are shown in Figure 4. It is clear that both E17F and E18R show excellent specificity. The B_max_ for E17F against *E. coli* O157:H7 is about 3.4x and 2.7x higher than the values against *E. coli* K12 and *E. coli* DH5α, respectively. Similarly, for E18R, the B_max_ is 16.7× and 5.4× higher than the values against *E. coli* K12 and *E. coli* DH5α, respectively.

Despite having high affinity, aptamers S1 and EcoR1 have relatively poor specificity. The high cross reactivity can be explained by the fact that neither S1 nor EcoR1 used a negative selection round against these strains of *E. coli* during the cell SELEX process [14,15]. Non-specific interaction was also reported in the original publications against these bacterial strains using dot blot assay [13].

As a result of epitope sharing among bacterial strains aptamers have high chances of binding non-specifically to other microorganisms [15], making their thorough evaluation important before incorporating into an assay, especially if the objective is to identify pathogenic serotype or strain among the same species of micro-organisms. As can be observed from the results obtained, all four aptamers show some degree of non-specific interaction with K12 and DH5α strains of *E. coli*, which is highly likely due to the epitope sharing in these bacterial strains. 

### 2.3. Effect of Biotin Tag on Aptamer Binding to E. coli O157:H7

**Figure 5:**
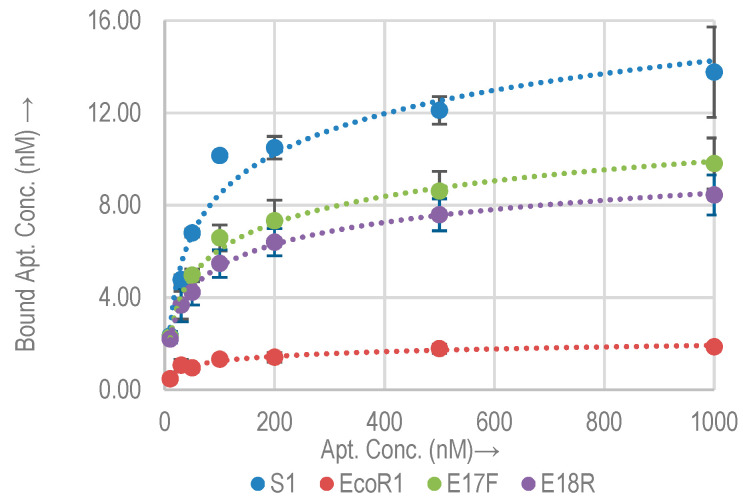
Binding isotherms for biotin tagged (5′) aptamers with *E. coli* O157:H7.

Chemical modifications can potentially affect the aptamer binding to their targets, which makes the evaluation of each modification important [31,32]. Previous research showed that the binding affinity of aptamers can change significantly by attachment of biotin tags. For example, in [24], aptamer (PA#2/8) selected for Protein A (a cell surface protein in gram positive bacteria) showed decreased binding upon 5′ biotinylation whereas the binding improved upon biotinylation at 3′. However, a 3′ biotin modification can potentially affect primer binding during amplification reactions. To avoid this only 5′ biotinylation modification was selected for all the aptamers under study, in order to evaluate the change in their binding capacities with *E. coli* O157:H7. Figure 5 shows the associated binding isotherms.

S1 showed a marked increase of about 2× in the binding capacity (Table 4), while the B_max_ decreased for all other aptamers. In the case of EcoR1, the B_max_ is reduced to 1.8 nM (0.5×), while as for E17F and E18R, B_max_ are 9.8 nM (0.3×) and 8.4 nM (0.15×) respectively. The B_max_ and K_d_ for all aptamers with a biotin tag are listed in Table 4 Kd and Bmax of aptamers with and without biotin tagged at 5′ end on E. *coli* O157:H7. Data represent Mean ± SD of 3 independent experiments in Table 4. K_d_ of EcoR1 with biotin tag was measured in [15] to be 41 nM based on indirect ALISA. In [15], the measurement was performed with extracted *E. coli* antigens coated in microtiter plates. In our study, aptamers were tested on the cultured whole cells of *E. coli* O157:H7 directly. Our measured K_d_ is comparable to the reported number.

Except S1, the biotin tag appears to hinder the binding of aptamers to the target sites. This is very important as most aptamers are selected through the SELEX or cell-SELEX process without the biotin tag, while they are often used in biosensors with a biotin tag [28,33]. The understanding of this discrepancy of binding is critical for the design of biosensors. 

### 2.4. Effect of Ionic Strength on Aptamer-Target Binding

The effect of ionic strength on aptamer-target binding was carried out using binding buffers of different mono valent and divalent ion concentrations. Six buffers, listed in Table 5, were tested. The first two buffers only contain Na^+^ and K^+^ ions without any Mg^2+^ ion. Buffers 3–6 contain increasing concentration of Mg^2+^ ions ranging from 0.5 mM (Buffer 3) to 10 mM (Buffer 6). The aptamer concentration was fixed at 500 nM (incubated with ≈10^8^ cells).

**Figure 6:**
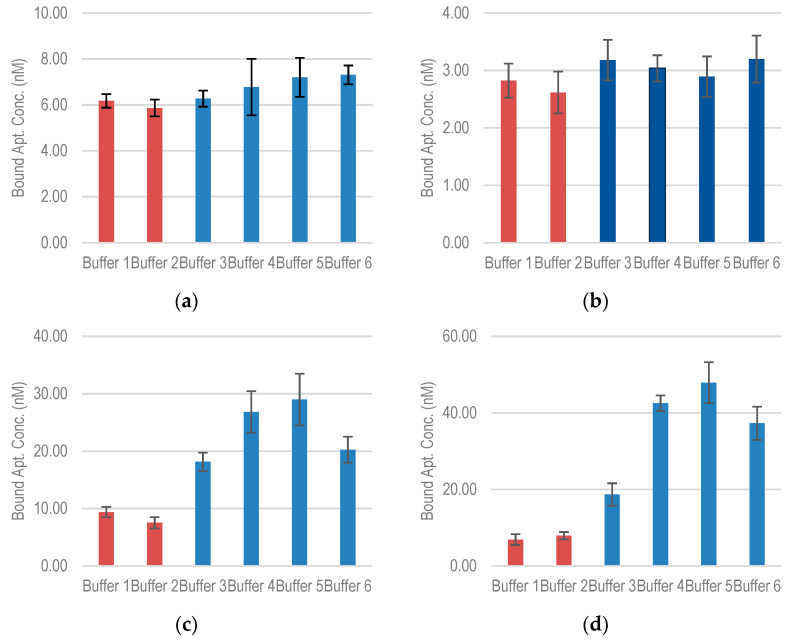
Bound aptamer concentration for (**a**) S1 (**b**) EcoR1 (**c**) E17F (**d**) E18R with *E. coli* O157:H7 in buffers of different ionic compositions (incubated aptamer concentration was fixed at 500 nM).

For aptamers S1 and EcoR1, the absence of Mg^2+^ was not found to have a significant effect on the binding capacity (Figure 6a,b). For S1, the addition of 5 mM K^+^ reduced the binding capacity by about 5%. As Mg^2+^ concentration is varied from 0.5 mM to 10 mM, the binding concentration increases by about 15%. In EcoR1, a similar effect was observed with the addition of K^+^, while the increase in Mg^2+^ does not cause any significant change in the binding capacity.

More significant changes were observed for E17R and E18R (Figure 6c,d). Without Mg^2+^, the binding capacity of E17R decreases drastically to about 9.3 nM. As Mg^2+^ concentration is increased, the binding capacity shows an upward convex trend with a maximum at 5 mM Mg^2+^ concentration (29 nM), following which, it starts to decrease. A similar trend is observed for E18R, in which the binding capacity decreases to 6.8 nM without Mg^2+^ and increases to a maximum of about 47 nM at 5 mM Mg^2+^ concentration. This trend suggests that Mg^2+^ initially helps screen the charge of the aptamer chain (negatively charged due to phosphate backbone), which allows binding to the negatively charged lipopolysaccharide targets. This screening is most effective at 5 mM. After that, it seems to hinder the binding.

It is generally well acknowledged that the presence of cations in the binding buffer can cause conformational changes in the aptamers and hence may result in a change in their binding characteristics. The presence of divalent cations is known to stabilize the aptamer secondary structure by screening the negative charge on the aptamer backbone [34]. The interaction between metal cations and the aptamer nucleic acid backbone, usually occurs either at the negatively charged phosphate group or the aromatic base in the aptamers. Alkali metals are capable of binding to both, while alkaline earth metals are known to interact preferably with the phosphate groups [21,35].

## 3. Materials and Methods

### 3.1. Bacterial Strains and Culture Conditions

The bacterial strain *E. coli* O157:H7 (ATCC 43888) was used as the target bacterial strain. It was cultured in tryptone soy broth (TSB) medium (CM0129, Oxoid, Hong Kong). Whole bacterial cells were cultured overnight at 37 °C with constant shaking, overnight culture was seeded into fresh TSB medium and cells were grown to an approximate concentration of 10^8^ CFU/mL. One ml bacterial culture in centrifuge tubes was washed twice by centrifugation (Hitachi Koki, Himac CT15E, Hong Kong) at 5500 rpm for 5 min using 1 × phosphate buffer saline (PBS–D8527, Sigma-Aldrich, Hong Kong). Washed bacterial cells were used for further binding study. *E. coli* K12 and DH5α were cultured in Luria–Bertaini (Affymetrix) media under the same growth conditions as given for *E. coli* O157:H7. These strains of *E. coli* were used for studying the non-specific binding of aptamers.

### 3.2. Aptamers and Primers

Five DNA based aptamers were selected from literature. These aptamers previously were selected specifically for *E. coli* O157:H7. The aptamer sequences and their primer sequences (as used in reported publications) are listed in Table 1**.** with their reported K_d_ values. All the aptamer and primer sequences were ordered from IDT Singapore. Before incubating with washed bacterial cells, the aptamers were dissolved in binding buffer (50 mM Tris-HCl-pH 7.5, 5 mM KCl, 50 mM NaCl, 1mM MgCl_2_) to a final volume of 200 μL in different concentrations. The dissolved aptamers were denatured by heating at 95 °C for 10 min and let to sit at room temperature for 30 min in order to allow them to renature into their secondary structures. As a control, an 80-base-pair random sequence (RS) was included in the binding experiments at the same conditions. Its sequence is also listed in Table 1.

### 3.3. Aptamer–E. coli Binding Assay

*Aptamer–E. coli binding—*Washed bacterial cells prepared as mentioned in Section 3.1 were incubated with different concentrations of renatured aptamers ranging from 1 nM to 1000 nM for 1 h at room temperature under constant agitation of 600 rpm (Thermomixer, Eppendorf, Hong Kong). Bacterial cells undergoing aptamer binding were washed three times by centrifugation at 5500 rpm for 5 min each with 1 × PBS. Thorough washing was carried out for 1 h per wash. To ensure proper washing to remove the unbound aptamers, bacterial pellets were dislodged using a Hula Mixer (Thermo Fischer, Scientific, Hong Kong). All binding assays were performed in triplicates independently.

*Elution of bound aptamers*—To elute bound aptamers from *E. coli*, cells were resuspended in 100 μL of DNase free water and heated at 95 °C for 10 min, followed by cooling on ice for 15 min. The cell suspensions were centrifuged at 13,000 rpm for 30 min to harvest bound aptamers in the supernatant. The supernatant was then transferred in a fresh microcentrifuge tube, followed by the addition of 0.3 M (final concentration) sodium acetate buffer pH 5.2 (S7899, Sigma-Aldrich, Hong Kong), and 3 volumes of chilled absolute ethanol (Sigma-Aldrich). The aptamers precipitation in ethanol was allowed to occur by storing at 4 °C overnight followed by centrifugation at 13,000 rpm for 20 min at 4 °C to pellet down the aptamers. Ethanol in the supernatant was drained off. Remaining salts were washed off by 70% ethanol (twice). Pelleted aptamers were air dried and resuspended in 50 μL DNase free water for qualitative and quantitative analysis.

*Negative Control*—The negative control is designed by processing bacterial cells that were not incubated with any aptamer following the same procedure. The pellets obtained from these cells were used as negative controls and put through both qualitative (PCR) and quantitative analysis (qPCR) along with cells from each binding experiment.

### 3.4. Qualitative Aptamer-Target Binding Analysis

*Polymerase chain reaction (PCR)—*PCR was carried out in a Veriti thermal cycler (Applied Biosystems, Hong Kong) in order to qualitatively analyse the binding of aptamers to the target cells. The assays were performed in 25 µL PCR reaction volume, containing 12 μL PCR master mix (RR300A, Takara, Beijing, China), 1 μL template (bound aptamers), 1 μL forward primer, 1 μL reverse primer from 5 µM stock, rest of the volume was made up with nuclease free water. The thermal cycling procedure starts with the initial denaturation for 5 min at 95 °C and is followed by 30 rounds of amplification. Each round of operation includes denaturation at 95 °C for 45 s, annealing at 55 °C for 45 s, extension at 70 °C for 45 s and final extension at 70 °C for 5 min.

*Gel electrophoresis*—Upon completion of PCR, the aptamer presence was checked on a standard 1.5% agarose gel, stained with pico green for visualization of amplicons. The gel was run in 1 × TAE (Tris acetic acid, EDTA disodium salt) buffer at 120 V for 40 min along with the 20 bp DNA marker (3420A, Takara). The gel was visualized on a gel documentation system (Bio-Rad’s Gel Doc XR+ system, Hercules, CA, USA).

### 3.5. Quantitative Aptamer-Target Binding Analysis (K_d_ Analysis)

Bacterial cells (≈10^8^ bacterial cells/ml) were incubated with aptamers, prepared in concentrations ranging from 0.1 nM to 1000 nM. Bound aptamers were recovered by ethanol precipitation after washing off the unbound aptamers. The quantification of aptamers bound to the cells was carried out by qPCR analysis (LightCycler 480 System, Roche Life Science, Basel, Switzerland) using SYBR Green I chemistry (04887352001, Roce).

Ten μL qPCR reaction was set up containing 1 μL template (bound aptamers), 5 μL of SYBR green qPCR Master mix (04887352001, Roche), 0.5 μL of 5 μM forward primer, 0.5 μL of 5 μM reverse primer, and 3 μL of nuclease-free water. The reactions were carried out in triplicates in 384 well plates. A melting curve analysis was performed from 55 °C to 85 °C to detect potential nonspecific products. The thermal cycling conditions followed were the same as those followed for PCR analysis. Calibration curves were used for data quantification by using known aptamer concentrations varying from 10^−2^ pmol to 10^−6^ pmol (Supplementary Figure S3). A separate calibration curve was plotted for all assays.

Saturation curves were plotted based on the qPCR data after the data was normalized against the negative control. The K_d_ and B_max_ of the aptamer were calculated by non-linear regression analysis.

### 3.6. Specificity of Aptamers

To evaluate the cross reactivity of the four aptamers with other *E. coli* strains, the binding assay was performed with *E. coli* K12 and DH5α. Both bacterial cells were cultured in LB media under conditions as described earlier. The four aptamers included in the study were incubated in the concentration ranges of 10 nM to 1000 nM, with bacteria (≈10^8^ bacterial cells). Binding analysis of the aptamers was done in a similar fashion using qPCR as is described for target *E. coli* cells.

### 3.7. Effect of Biotin-Tag Modification on the Binding Efficiency of Aptamers

An analysis was set up to study the effect of the biotin tag on the binding affinity of the aptamers to *E. coli* O157:H7. All the four aptamers under study were modified with biotin at the 5′ terminus. The modified aptamers were directly ordered from IDT Singapore. *E. coli* O157:H7 cells (≈10^8^ bacterial cells) were incubated with different concentrations of aptamers ranging from 10 nM to 1000 nM. The rest of the protocol for binding was similar to that given in above.

### 3.8. Effect of Ionic Strength on the Aptamer-Target Binding

The effect of mono and dibasic salt ion strength on the binding characteristics of aptamers was analyzed by changing the concentration of KCl and MgCl_2_ in the binding buffer. Five hundred nM aptamers were incubated with bacterial culture grown (≈10^8^ bacterial cells). A total of six binding buffers in two sets as shown in Table 5 were prepared for this study.

## 4. Conclusions

In this paper, we presented a comprehensive study of DNA-based aptamers for *E. coli* O157:H7. Five DNA aptamers selected from the literature were compared. Among the five aptamers compared, AM6 was excluded from the study as its primer showed non-specific binding to *E. coli* genomic DNA. Aptamer E18R showed the highest binding capacity (B_max_) to *E. coli* O157:H7, while EcoR1 showed the lowest dissociation constant (K_d_). E18R showed the lowest binding capacity to other strains of *E. coli* compared to *E. coli* O157:H7 (maximum specificity).

Our study also showed that aptamer binding characteristics could be affected significantly with the biotin tag. With a biotin modification at the 5′ end of the aptamer, binding capacities were seen to decrease in all the selected aptamers, except S1. The difference in ionic strength of the binding buffer was also evaluated. Mg^2+^ was found to be critical for the binding of E17F and E18R, while S1 and EcoR1 were not sensitive to Mg^2+^.

## Figures and Tables

**Table 1:** Aptamers analyzed in this study with its primers.

	bp	Reported K_d_	Sequence	Primers
AM6 [13]	117	107.6 ± 67.8 pmol	CGTGATGATGTTGAGTTGGGGTGATGGGTGCATGTGATGAAAGGGGTTCGTGCTATGCTGTTTTGTCTAATAATACTAGTCCTTGCCAAGGTTTATTCCAGTAATGCCAACCAATCT	FP-
				CGTGATGATGTTGAGTTG RP-
				AGATTGGTTGGCATTACTG
S1 [14]	90	10.30 nM	CAGTCCAGGACAGATTCGCGAGTGGTCGTGGTGAGGTGCGTGTATGGGTGGTGGATGAGTGTGTGGCCACGTGGATTTCATTCAGCGATT	FP-CAGTCCAGGACAGATTCGCGAG
				RP-AATCGCTGAATGAAATCCACGTG
EcoR1 [15]	88	41 ± 2 nM	ATTAGTCAAGAGGTAGACGCACATATCATCACAGCCGCAGCCGCCCCTTCCATTCACATGCCAGCTTCTGGTCGTCGTGACTCCTATA	FP-ATAGGAGTCACGACGACCAGAA RP-ATTAGTCAAGAGGTAGACGCACATA
E17F [25]	72		ATCCGTCACACCTGCTCTATCAAATGTGCAGATATCAAGACGATTTGTACAAGATGGTGTTGGCTCCCGTAT	FP-
				ATCCGTCACACCTGCTCT RP-
				ATACGGGAGCCAACACCATC
E18R [25]	72		ATACGGGAGCCAACACCATTCTATCGTTCCGGACGCTTATGCCTTGCCATCTACAGAGCAGGTGTGACGGAT	FP-
				ATACGGGAGCCAACACCA RP-
				ATCCGTCACACCTGCTCT
RS	80		ATCCAGAGTGACGCAGCATGCTTAAGGGGGGGGCGGGTTAAGGGAGTGGGGAGGGAGCTGGTGTGGACACGGTGGCTTAGT	FP-
				ATCCAGAGTGACGCAGCA RP-
				ACTAAGCCACCGTGTCCA

**Table 2:** Measured dissociation constant (K_d_) and binding capacity (B_max_) values for the four selected aptamers for *E. coli* O157:H7 and their comparison with the B_max_ of the RS. Data represent Mean ± SD of 3 independent experiments.

Aptamer	K_d_ (nM)	B_max_ (nM)	Binding Sites Per Bacteria	B_max_ Compared to RS
S1	25.7 ± 12	6.61 ± 1.1	1980	10×
EcoR1	45.5 ± 17	3.42 ± 0.5	1030	5×
E17F	135 ± 72	30.17 ± 4.4	9050	47×
E18R	151 ± 80	54.30 ± 10	16,290	85.64×

**Table 3:** Aptamer binding capacity (B_max_) with nonspecific *E. coli*. Data represent Mean ± SD of 3 independent experiments.

Aptamer	*E. coli* K12			*E. coli* DH5α		
	K_d_ (nM)	B_max_ (nM)	Approx. Binding Sites/ Bacteria	K_d_ (nM)	B_max_ (nM)	Approx. Binding Sites/ Bacteria
S1	62.9 ± 27	6.41 ± 0.1	1920	114.2 ± 23	3.16 ± 0.1	940
EcoR1	75.2 ± 28	5.36 ± 0.1	1600	48.3 ± 24	2.45 ± 0.1	730
E17F	202 ± 116	8.78 ± 0.6	2630	87.7 ± 14	11.2 ± 0.4	3360
E18R	111.3 ± 49	3.54 ± 0.2	1060	95.5 ± 24	11.0 ± 0.5	3300

**Table 4:** K_d_ and B_max_ of aptamers with and without biotin tagged at 5′ end on *E. coli* O157:H7. Data represent Mean ± SD of 3 independent experiments.

Aptamer	with Biotin		without Biotin	
	K_d_ (nM)	B_max_ (nM)	K_d_ (nM)	B_max_ (nM)
S1	25.7 ± 12	13.76 ± 1.94	57.6 ± 10.6	6.61 ± 1.1
EcoR1	45.5 ± 17	1.85 ± 0.03	33.7 ± 2.5	3.42 ± 0.5
E17F	135 ± 72	9.8 ± 0.9	51.2 ± 5.3	30.17 ± 4.4
E18R	151 ± 80	8.44 ± 0.5	45.5 ± 3.7	54.30 ± 10

**Table 5:** Binding buffers with different ionic strengths.

Binding Buffer without Dibasic Salt Ions
Buffer 1—50 mM Tris-HCl Ph 7.5, 50 mM NaCl.
Buffer 2—50 mM Tris-HCl pH 7.5, 50 mM NaCl, 5 mM KCl.
**Binding Buffer with Dibasic Salt Ions**
Buffer 3—50 mM Tris-HCl pH 7.5, 50 mM NaCl, 5 mM KCl, 0.5 mM MgCl_2_.
Buffer 4—50 mM Tris-HCl pH 7.5, 50 mM NaCl, 5 mM KCl, 1 mM MgCl_2_.
Buffer 5—50 mM Tris-HCl pH 7.5, 50 mM NaCl, 5 mM KCl, 5 mM MgCl_2_.
Buffer 6—50 mM Tris-HCl pH 7.5, 50 mM NaCl, 5 mM KCl, 10 mM MgCl_2_.

## Data Availability

The data presented in this study are available on request from the corresponding author.

## Supplementary Materials

The following are available online, Figure S1: Minimum free energy structures predicted by mfold softwareat 37 °C(binding buffer conditions were similar to the onesused in this study)for aptamers (a) AM6 (b) S1(c) EcoR1(d) E17F(e)E18R. Free energies predicted by the software for these structures from a–e are, −5.32 kcal/mol, −6.47 kcal/mol, −3.12 kcal/mol, −4.56 kcal/mol, −4.49 kcal/mol, respectively, Figure S2: Using Multiple Sequence alignment software MUSCLE by Ensembl, 5 aptamer sequences were analysedfor sequence homology to find any conservative motifs present, Figure S3: Representative calibration curves obtained using known aptamer concentrations (10^−6^–^10−2^ pmoles) for the E.coli O7:H157 case (a)S1 (b)EcoR1 (c)E17F (d)E18R. Separate calibration curves were obtained for the other E.coliand aptamer combinations, Figure S4: Amplification curves obtained *E.coli* O7:H157 case (a)S1 (b)EcoR1 (c)E17F (d)E18R. The standard (single value) as well as bound aptamer amplification curves(triplicates) are shown here.
