# Supplementary material for: Binding Characteristics Study of DNA based Aptamers for E. coli O157:H7
Source: Molecules. 2021 Jan 3;26(1):204. doi: 10.3390/molecules26010204 (PMC7795876; doi:10.3390/molecules26010204)
Supplement: Supplementary file 1 [file molecules-26-00204-s001.pdf]

Supplementary Materials

Binding Characteristics Study of DNA based Aptamers for *E. coli* O157:H7

Saika Siddiqui [1*] and Jie Yuan[2]

[1] Dept. of Bioengineering, Hong Kong University of Science and Technology, Kowloon, Hong Kong; ssiddiqui@connect.ust.hk

[2] Dept. of Electronic and Computer Engineering, Hong Kong University of Science and Technology, Kowloon, Hong Kong; eeyuan@ust.hk

* Correspondence: ssiddiqui@connect.ust.hk

SUPPLEMENTARY FIGURES

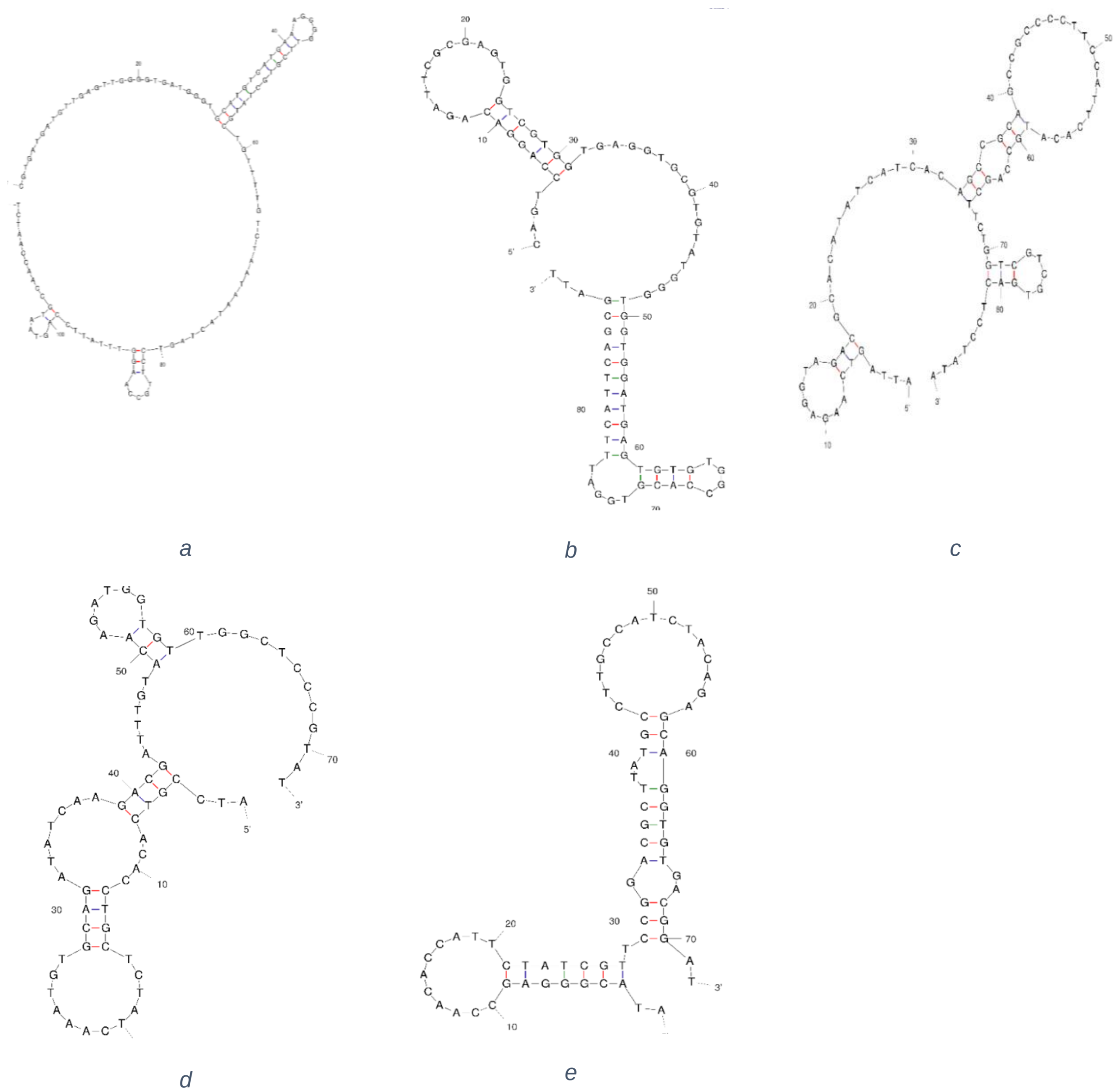

Supplementary Figure S1:

Minimum free energy structures predicted by mfold software at 37°C (binding buffer conditions were similar to the ones used in this study) for aptamers (a) AM6 (b) S1(c) EcoR1(d) E17F(e) E18R. Free energies predicted by the software for these structures from a-e are, -5.32kcal/mol, -6.47kcal/mol, -3.12kcal/mol, -4.56kcal/mol, -4.49kcal/mol, respectively.

```
CLUSTAL multiple sequence alignment by MUSCLE (3.8)


AM6             CGTGATGATGTTGAGTTGGGGTGATGGGTGCATGTGATGAAAGGGGTTCGTGCTATGCTG
E18R            -------------------ATACGGGAGCCAACACCATTCTATCGTTCCGGACGCTTATG
S1              ------------CAGTCCAGGACAGATTCGCGAGTGGTCGTGGTG--AGGTGCGTGTATG
E17F            ---------ATCCGTC---------ACACCTGCTCTATCAAATGTGCAGATATCAAGACG
EcoR1           ---------ATTAGTCAAGAGGTAGACGCACATATCATCACAGCCGCAGCCGCCCCTTCC
                                                     *

AM6             TTTGTCTAATAATACTAGTCCTTGCCAAGGTTTATTCCAGTAATGCCAACCAATCT
E18R            CCTTGCCATCTACAGAGCAGGTGTGACGGAT-------------------------
S1              GGTGGT------GGATGAGTGTGTGGCCACGTGGATTTCATTCAGCGATT-------
E17F            ATTTGT--------------ACAAGATGGTGTTGGCTCCCGTAT-------------
EcoR1           ATTCAC------ATGCCAGCTTCTGGTCGTCGTGACTCCTATA-------------
                  *                     *
```

Supplementary Figure S2:

Using Multiple Sequence alignment software MUSCLE by Ensembl, 5 aptamer sequences were analysed for sequence homology to find any conservative motifs present.

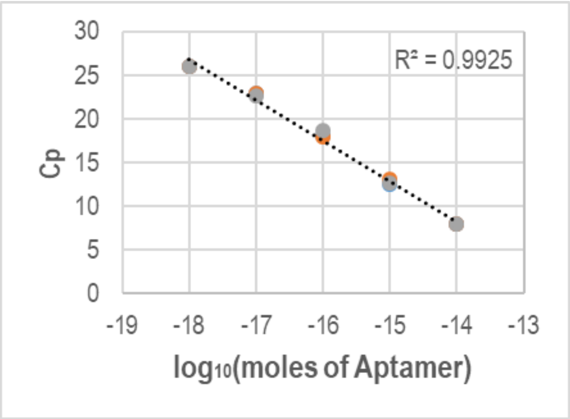

(a)

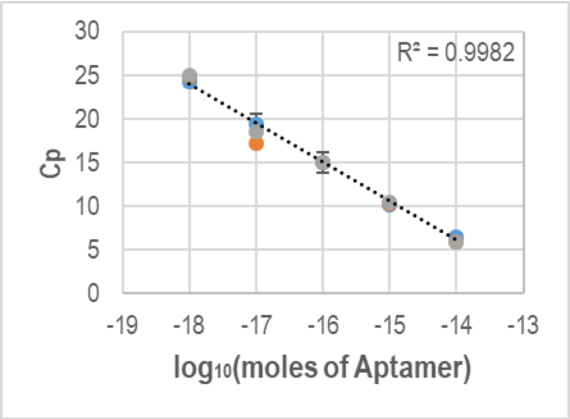

(b)

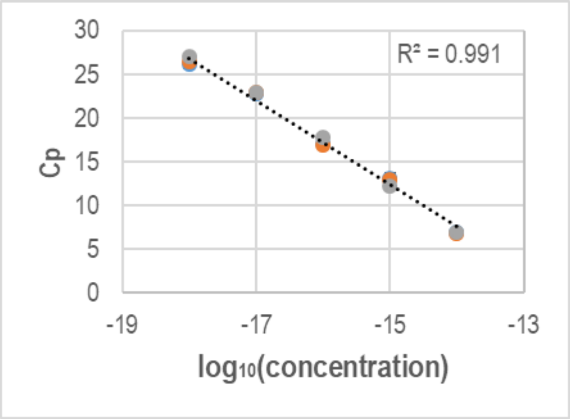

(c)

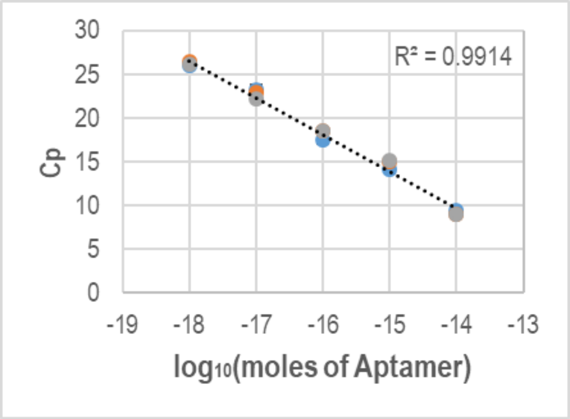

(d)

Supplementary Figure S3:

Representative calibration curves obtained using known aptamer concentrations (10^{-6}–10^{-2} pmoles) for the *E. coli* O7:H157 case (a)S1 (b)EcoR1 (c)E17F (d)E18R. Separate calibration curves were obtained for the other *E. coli* and aptamer combinations.

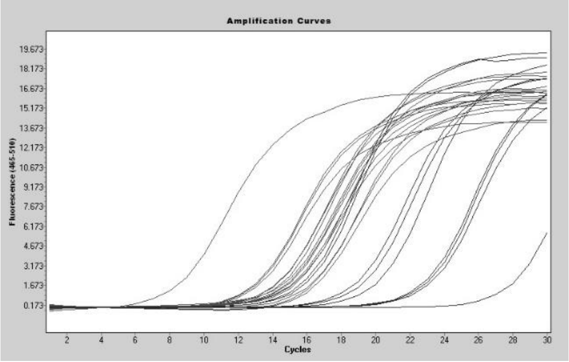

(a)

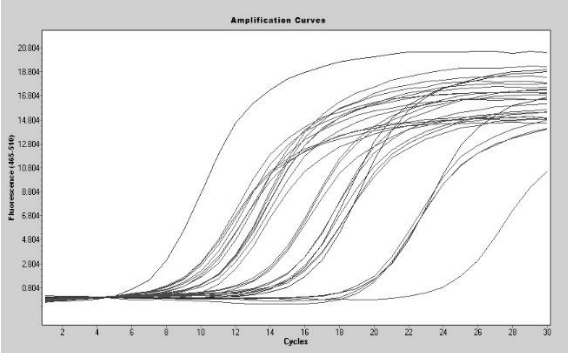

(b)

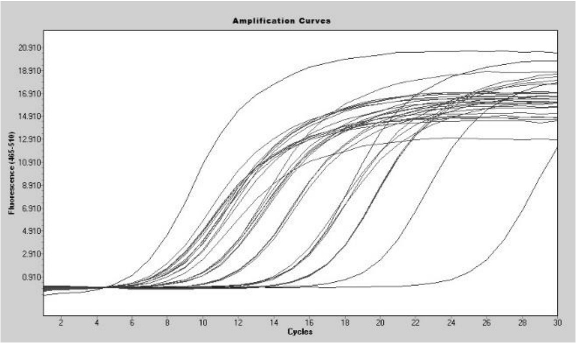

(c)

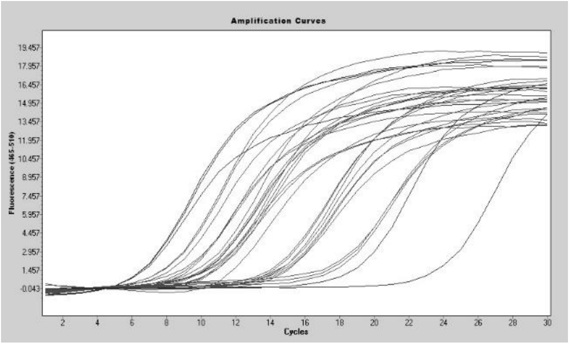

(d)

Supplementary Figure S4:

Amplification curves obtained *E. coli* O7:H157 case (a)S1 (b)EcoR1 (c)E17F (d)E18R. The standard (single value) as well as bound aptamer amplification curves (triplicates) are shown here.
